# Future Directions in Reducing Gastrointestinal Disorders in Children With ASD Using Fecal Microbiota Transplantation

**DOI:** 10.3389/fcimb.2021.630052

**Published:** 2021-02-26

**Authors:** Paulina Żebrowska, Izabela Łaczmańska, Łukasz Łaczmański

**Affiliations:** ^1^Laboratory of Genomics and Bioinformatics, Hirszfeld Institute of Immunology and Experimental Therapy, Polish Academy of Sciences, Wrocław, Poland; ^2^Department of Genetics, Wroclaw Medical University, Wrocław, Poland

**Keywords:** autism spectrum disorder, fecal microbiota transplantation, inflammatory bowel disease, gastrointestinal symptoms, therapy

## Abstract

Research on the use of fecal microbiota transplantation (FMT) in the treatment of disorders related to digestive system ailments in children with autism spectrum disorders (ASDs) is a new attempt in a therapeutic approach. There are very little scientific evidences available on this emerging alternative method. However, it appears to be interesting not only because of its primary outcome, relieving the gastrointestinal (GI) symptoms, but also secondary therapeutic effect of alleviating autistic behavioral symptoms. FMT seems to be also promising method in the treatment of another group of pediatric patients, children with inflammatory bowel disease (IBD). The aim of this study is to discuss the potential use of FMT and modified protocols (MTT, microbiota transfer therapy) in the treatment of GI disorders in ASD children supported by reports on another disease, IBD concerning pediatric patients. Due to the few reports of the use of FMT in the treatment of children, these two patients groups were selected, although suffering from distant health conditions: neurodevelopmental disorder and gastrointestinal tract diseases, because of the the fact that they seem related in aspects of the presence of GI symptoms, disturbed intestinal microbiota, unexplained etiology of the condition and age range of patients. Although the outcomes for all are promising, this type of therapy is still an under-researched topic, studies in the group of pediatric patients are sparse, also there is a high risk of transmission of infectious and noninfectious elements during the procedure and no long-term effects on global health are known. For those reasons all obtained results should be taken with a great caution. However, in the context of future therapeutic directions for GI observed in neurodevelopmental disorders and neurodegenerative diseases, the topic seems worthy of attention.

## Introduction

Fecal microbiota transplantation (FMT) is proposed as an alternative therapeutic approach for the treatment of recurrent *Clostridium difficile* (rCDIs) (Rao and Safdar, 2016) that cause injury to the gastrointestinal (GI) tract and diarrhea (Kuijper et al., 2006). The Food and Drug Administration (FDA) has not approved the use of FMT as a treatment for nonresponsive *C. difficile* infections (CDIs) however patients can benefit from access to this type of treatment under the policy of “enforcement discretion” (U.S. Food and Drug Administration, [Accessed May 22, 2020]). In clinical practice, FMT or microbiological replacement therapy is now used solely in the treatment of rCDIs (McDonald et al., 2018) and restricted to patients not responding positively to standard treatment procedures (Tan and Johnson, 2019). Its use in other diseases is experimental and still under research (Gupta et al., 2020). Nonetheless, interest in this form of therapy has shifted from infectious to noncommunicable diseases associated with the GI tract and also other disorders (Wang et al., 2019a). Currently, much of this interest is focused on the use of FMT in treating inflammatory bowel disease (IBD) and functional bowel disorders such as irritable bowel syndrome, as well as obesity, metabolic syndromes, multiple sclerosis, and colon cancer and most importantly, GI symtoms in autism spectrum disorders (ASDs) and other neurological disorders (Garrett, 2019; Wang et al., 2019a; Vendrik et al., 2020).

The human microbiota is a large community consisting mainly of bacteria, archaea, eukaryotic microorganisms, and viruses that share a specific environment—a human body (Lederberg and McCray, 2001; Turnbaugh et al., 2007; Wang et al., 2017). Beside the nuclear and mitochondrial genomes the microbiome, which refers to the genetic material of the personal microbiota, plays an important role in the human health and disease (Wang et al., 2017), for example by complementing molecular and metabolic functions (Shafquat et al., 2014). In particular, the gut microbiome is seen as an essential element ensuring homeostasis (Sekirov et al., 2010; Boem and Amedei, 2019). Moreover it has been reported that enteric and central nervous systems can communicate and interact with the immune, endocrine, and neuronal systems as inherent elements of influence (Dinan and Cryan, 2017). The concept of the gut–microbiota–brain axis as a two-way communication system using multiple common ways like autonomic nervous system and production of neurotransmitters or short–chain fatty acids (SCFAs) (Scriven et al., 2018), has gained favor over the years in an ecosystem approach to the health (Srikantha and Mohajeri, 2019). The interest in this subject continous to grow and is thoroughly described (Scriven et al., 2018; Cryan et al., 2019; Garcia-Gutierrez et al., 2020) due to its proven role in regulating a number of body functions (Boem and Amedei, 2019).

The crucial role of intestinal microbiota has been demonstrated in many health conditions, including allergy, disorders of internal secretion, complications related to nutritional status and metabolic changes such as obesity and diabetes, IBD, and also in autism (Shanahan, 2012).

Microbiota, as a nongenetic heritable element, contributes to metabolism and affects the nervous and immune systems of the host (Cho and Blaser, 2012; Sandoval-Motta et al., 2017; Pulikkan et al., 2019). Through gestation, during the labor, and after the birth, an infant inherits the vaginal, skin, fecal, and mouth microbiota of the mother (Jiménez et al., 2008; Satokari et al., 2009; Wopereis et al., 2014). Then, by means of the mother’s milk or formula, the newborn is supplied with another inoculum of microorganisms that will colonize its gut (Martín et al., 2003; Makino et al., 2011; Wopereis et al., 2014). The intestinal microbiota is formed in the first years of a child’s life (Wopereis et al., 2014). Some evidences shows that nearly half of microbiota maturity is reached around the age of three (Yatsunenko et al., 2012), and other indicates that microbiota maturation may last through adolescence (Agans et al., 2011; Ringel-Kulka et al., 2013). During this period, environmental factors, such as the child’s feeding type, breastfeeding status followed by diet dependent on culture and season, and another environmental exposures or hygiene, affect its formation (Lozupone et al., 2012; Wopereis et al., 2014). At this time, it can be easily perturbed, due to the use of corticosteroids or acid suppressants, eradicated by antibiotic and changed by probiotic therapies, or disturbed by infections or GI diseases (Navarro et al., 2016).

Dysbiosis, the imbalance of intestinal microbiota, is observed not only in the diseases related to the GI tract but also indirectly in gut-related complaints and nervous system disorders such as ASDs (Borre et al., 2014; Carding et al., 2015; Srikantha and Mohajeri, 2019). ASD refers to a heterogeneous group of dysfunctions that manifest as deficits in social relations, communication, and language development, as well as restricted interest and a repetitive pattern of behaviors (World Health Organization, 2019), frequently accompanied by comorbid GI symptoms (McElhanon et al., 2014). There is also a positive correlation between the exacerbation of neurological symptoms and GI inconvenience (Adams et al., 2011). ASDs are diagnosed in about 1% of children worldwide, yet no remedy has been found for this set of conditions (Wang et al., 2019b). Moreover a 10% increase in the prevalence of ASDs from 16.8 cases per 1,000 children in 2014 (Baio et al., 2018) compared to 18.4 cases per 1,000 children in 2016 (Maenner et al., 2020) has been detected.

IBD consists of two GI diseases, Crohn’s disease (CD) and ulcerative colitis (UC) (Malik, 2015). These entities are characterized by chronic and nonspecific inflammation, and also dysbiotic changes in composition of microbiota and reduction of its diversity (Kostic et al., 2014), which seems to be relevant in the progression of this disease (Fang et al., 2018). In connection with the growing awareness of the importance of gut microbiota (GM) in the pathogenesis of GI diseases, GM related therapies are of great interest in IBD treatment (Moutinho et al., 2019).

The gut dysbiosis in ASD is well documented; nonetheless, there is no defined microbial signature for autistic gut yet (Fattorusso et al., 2019; Andreo-Martínez et al., 2020). The main microbial shifts explored thus far in ASD are increased quantity and decreased diversity of microbiota, which are the factors favoring the overgrowth of pathogenic strains (Finegold et al., 2012; Kang et al., 2013; Fattorusso et al., 2019). There are observed significant changes in the composition of intestinal microbiota in children with ASDs compared to healthy controls (Liu et al., 2019a). This analysis showed that the group of patients with ASD had higher abundance of *Lactobacillus*, *Bacteroides*, *Desulfovibrio*, and *Clostridium* and lower abundance of *Bifidobacterium*, *Blautia*, *Dialister*, *Prevotella*, *Veillonella*, and *Turicibacter* (Liu et al., 2019a). Another systematic review also indicates such associations in people with ASDs compared to neurotypical controls—higher abundance of *Faecalibacterium* and *Lactobacillus* bacteria, as well as slightly increased numbers of *Ruminococcus* and *Clostridium* and reduced numbers of *Akkermansia*, *Bacteroides*, *Bifidobacterium*, *Escherichia coli*, and *Enterococcus* (Xu et al., 2019). Also the study of Martínez-González and Andreo-Martínez (2019) on differences in GI symptoms between children with ASDs and healthy controls, with a correlation analysis between GI symptoms and the number of intestinal bacteria revealed that the ASD group with comorbid GI symptoms showed higher abundance of *Candida* yeast, *Prevotella*, and *Streptococcus*, and lower abundance of *Veillonella*. Moreover, published in 2020 paper about gut microbiota profiling in ASD children displays higher abundance of *Roseburia* and *Candidia* genera and lower abundance of *Dialister*, *Bilophila*, *Coprococcus* and confirmes lower abundance of *Veillonella*, *Streptococcus* and *Prevotella* genera (Andreo-Martínez et al., 2020). The authors of all the above mentioned papers emphasize that it is still too early to conclude about the composition of the intestinal microbiota that may be correlated with ASDs. Research should be carried out on possibly homogeneous groups, in terms of age and the severity of neurological symptoms, taking into account the multidisciplinary approach to the problem of gut microbiome in ASD. Unified next-generation sequencing (NGS) approach paves the way for accurate analysis of microbiome by testing the microbiota composition with the use of 16S rRNA gene analysis. Due to the fact that GI symptoms severity correlates with severity of neurological symptoms of ASD (Adams et al., 2011; Chaidez et al., 2014) and that the level of GI disorders is in relation to GM alterations (Saurman et al., 2020)—the diversity and quantity, and enrichment or deficiency of some taxonomic groups, the present knowledge suggests that GM alterations may be associated with ASDs.

Abnormal fecal microbiota in both autistic children with comorbid GI disorders and patients suffering from IBD may suggest the correlation between intestinal dysbiosis due to inflammation and GI disturbances (Fattorusso et al., 2019). As one aspect of this hypothesis, the inflammatory process may be the result of abnormal GM. What was described in the study of Walker et al. (2013) were the authors compared the transcriptomic and the metagenomic profile from the small and large intestines from ASD, CD and UC patients hypothesize. The molecular profile of the gastrointestinal mucosa of children with ASD and GI symptoms coincides with that characteristic of IBD, but it has features that distinguish it (Walker et al., 2013) Another aspect of this hypothesis is the relation of gut dysbiosis and the the mucous barrier damage, causing an increase in the permeability of the intestinal epithelium (Santocchi et al., 2016), making children with ASD more sensitive to peptides of exogenous origin like those derived from the diet, and neurotoxic peptides of bacterial origin (de Magistris et al., 2010; Gonzalez et al., 2011) or the lipopolysaccharide (LPS) which elicits a pro-inflammatory response (Jyonouchi et al., 2002). However, results on intestinal permeability are inconclusive (Navarro et al., 2016). On the other hand, higher rate of enetrocyte damage is reported in ASD children with severe maladapitve behavior, compared to mild maladapitve behavior and control group (Pusponegoro et al., 2015). One of the possible effects of the response to increased passage of exogenous peptides through damaged epithelium may be the production of pro-inflammatory cytokines and their effects on the CNS (Santocchi et al., 2016). The relation between the microbiota and autism spectrum disorders has more aspects and attempts to explain their relationship, such as impact of SCFAs (Macfabe, 2012; MacFabe, 2015), bacterial neurotransmitters like GABA (Foss-Feig et al., 2017) hypothalamic–pituitar–adrenal (HPA) axis and communication *via* vagus nerve (Fülling et al., 2019), immune system activation (Rose et al., 2018), or serotonin pathway (Cryan et al., 2019) the discussion of which goes beyond the scope of this paper.

Therapeutic modulation of the intestinal microbiota, through various types of interventions, may influence the appearance, course and progression of neurological disorders (Vendrik et al., 2020). In the light of the latest scientific evidence, FMT, an emerging microbial replacement therapy, is successfully applied in the treatment of diseases characterized by altered GM (Gupta et al., 2020). FMT in the ASD with coexisting GI disorders is a novel therapeutic approach. Research in this area, however sparse, has been compiled here using this approach in the treatment of pediatric neurotypical IBD patients, due to the similarity of GI symptoms and age. A search of the literature on the use of FMT among pediatric patients suffering from inflammatory bowel disease was performed on the NCBI platform, in the MeSH (Medical Subject Headings) database with the query “Fecal Microbiota Transplantation” and “Inflammatory Bowel Diseases”. The results were pre-filtered using the variant “Child: birth-18”. According that 32 records were identified. Ten articles, case reports and open-label trials describing the clinical application of this method in patients were selected for further analysis. Similarly, the database was searched for articles on autism spectrum disorders. The search parameters were “Fecal Microbiota Transplantation” and “Autism Spectrum Disorder”, without any additional filter. Ten results were obtained, of which one article describing open-label clinical trial was included. Another article was incorporated by manual search. The inclusion criterion was the availability of the full text of the article. Review articles were not included in both cases.

## Gastrointestinal Disorders in ASD

In addition to the core neurological symptoms of ASDs, various concurrent symptoms such as seizures, psychiatric illness, intellectual disability, sleep disruption, and above mentioned feeding difficulties and GI disturbances occur, as suggested by prominent evidence that autism is not just a psychiatric disorder but may also have a physiological base (Pulikkan et al., 2019). Accumulating evidence shows that ASD children suffer from GI disorders more frequently than neurotypical children (Martínez-González and Andreo-Martínez, 2019). Common GI conditions such as constipation, diarrhea, and abdominal pain are correlated with the severity of ASDs (Adams et al., 2011).

The GI system has been implicated in the hypotheses of ASDs, both therapeutic and causal (Buie et al., 2010), as autistic children show a higher prevalence of GI distress compared to control children (McElhanon et al., 2014). Nearly half of the children with ASDs experience one or more chronic GI complaints (Kang et al., 2014) This type of ailments can hinder therapy and above all cause suffering that might be unnoticed and omitted by medics. Children with limited communication skills have a problem with expressing pain, and this can intensify or contribute to additional maladaptive behaviors considered as a manifestation of discomfort such as dyssomnia, hostile attitude or violent behavior, and self-mutilation (Buie et al., 2010).

The precise causality of GI distress is has not been known yet, and the etiology of autism is still unclear. However, it has been hypothesized that GI symptoms in autism have a functional, rather than an organic origin, which means that the condition results from behaviors, medications, or dietary habits (Ibrahim et al., 2009; Gorrindo et al., 2012). On the other hand, a recently published retrospective study of pediatric patients undergoing colonoscopy showed that of the 50 patients qualified for the diagnosis of eosinophilic colitis (EC), 22 of them were ASD children (the prevalence of ASD in the group of patients with EC was 44%) (Raffaele et al., 2021). This is another justification for the selection of the comparator group, because IBD, also infections with *Salmonella*, *E. coli*, *H. pylori*, and *C. difficile* (apart from parasitic infections, which are frequent in children), cause hypereosinophilia (Rogowska, 2012; Raffaele et al., 2021). Bearing in mind that the cause-and-effect chain in life sciences is neither one-way nor linear (Boem and Amedei, 2019), in addition to genetic factors that may rudiment this group of disorders (de la Torre-Ubieta et al., 2016; Feliciano et al., 2019; Łaczman´ska et al., 2020), environmental factors such as gut microbiota seems to be relevant (Pulikkan et al., 2019). Therefore, there is a hope that the GI symptoms can be eliminated if we know their cause. This in turn can be made possible by testing the microbiome on a large scale. The microbiome analysis and therapies aimed at rebalancig the microbiota can help parents and caregivers of these children in their day-to-day care and development.

Considering the difficulties in quantitative assessment, the median of prevalence proportion of GI complaints detected in various studies among people with ASDs has been calculated, which is 46.8% for any or aggregation of more than one GI symptom, 22% for constipation, 14% for abdominal discomfort, and 13% for diarrhea (Holingue et al., 2018). It has been estimated that approximately 95% of childhood constipation may not have a primary physiological cause but a functional one (Pashankar, 2005). Many children with ASDs present additionally enhancing conditions, such as delayed learning or lack of toilet skills, which negatively affect bowel emptying (D’Cruz et al., 2013). Children with ASDs also have difficulties in processing sensory stimuli, as well as often exhibit limited mobility resulting from their pattern of behaviors, which are secondary factors that can reduce GI motility (Peeters et al., 2013).

It is important to standardize measurement approaches to more precisely estimate the prevalence of GI disturbances. This should be done for a better understanding of the potential risk factors within the GI tract and their role in the development of ASD symptoms. Another important aim is to assess the safety and potency of the interventions on GI symptoms (Holingue et al., 2018). Nevertheless, the GI problems among ASD people are evident and require treatments.

## Microbial-Mediated Therapeutic Approaches in ASD with Comorbid GI

There are three known approaches to restore the ecological balance in the gut affected by dysbiosis: 1) probiotic therapy, which is inoculation with beneficial bacteria strains, 2) prebiotic supplementation, i.e. providing nutrients that promote the growth of beneficial bacteria, or 3) bacteriotherapy consisting in transplanting the entire ecosystem, considered as healthy (Lozupone et al., 2012). The question that has remained unanswered for years is which strategy will be reliable, safe and best for the individual patient (Lozupone et al., 2012). There are several approaches that seem to be promising not only in the treatment of GI symptoms among people with ASDs but also in alleviating behavioral symptoms (Saurman et al., 2020).

Antibiotic therapy is an approach that disturbs existing microbial balance by suppressing growth of particular components of that ecosystem. An extremely important step in supporting the hypothesis that changes in GM can cause changes in the core symptoms of ASD were the results of the study by Sandler et al. (2000) taking into account the involvement of Clostridiales as a factor associated with the occurrence of regressive autism (Saurman et al., 2020). It was then shown that as a result of the use of vancomycin against the neurotoxic compounds–producing Clostridiales, some ASD symptoms temporarily alleviated, only to return after discontinuation of this antimicrobial agent (Sandler et al., 2000).

Administration of probiotics aiming to restore gut microbial balance also seems to be promising (Saurman et al., 2020). The recent data on the therapeutic use of probiotics in ASDs revealed suggestive evidence and improvement trend in the behavioral and GI symptoms after the administration of these supplements (Liu et al., 2019b; Martínez-González and Andreo-Martínez, 2020).

Dietary interventions are known as modulating factors of the intestinal microbiota (Jandhyala et al., 2015); hence, diet changes are considered as a far-reaching approach due to the relief of ASD symptoms (Saurman et al., 2020).

All these approaches may not be effective therefore FMT was proposed as a promising procedure in comorbid GI.

## FMT Procedure Based on the Use in Pediatric IBD

The FMT procedure involves collecting stool from a healthy selected donor and transplanting it to the prepared recipient’s GI tract in order to restore dysbiotic intestinal microbiota and thereby promote healthiness (Gupta and Khanna, 2017; Wang et al., 2019a). There is no standard procedure established so far. The preparation of the recipient may involve administration an antibiotic to facilitate colonization with the donor bacteria (Goyal et al., 2018; Cho et al., 2019) or preparation requires the discontinuation of the use of the antibiotic before the procedure (Hourigan et al., 2015; Karolewska-Bochenek et al., 2018). It may also involve colonic lavage (Shimizu et al., 2016). The specimen used in the FMT procedure is fresh (Hourigan et al., 2015; Vandenplas et al., 2015; Nusbaum et al., 2018; Moutinho et al., 2019) or frozen stool preparation (Karolewska-Bochenek et al., 2018; Nusbaum et al., 2018). The procedure consists of screening donors and material for known pathogens. The donor can be age-related (Vandenplas et al., 2015) or not (Nusbaum et al., 2018), an unrelated person (Goyal et al., 2018; Karolewska-Bochenek et al., 2018; Nusbaum et al., 2018), a relative (Vandenplas et al., 2015; Shimizu et al., 2016; Nusbaum et al., 2018; Moutinho et al., 2019), or a donor from a stool bank (Cho et al., 2019). There are several possible routes of administration; upper digestive tract *via* nasogastric or nasoenteric tubes and upper endoscopy (gastroscopy), infusion through the lower digestive system, enema, colonoscopy or sigmoidoscopy (van Nood et al., 2013a; Youngster et al., 2014; Cammarota et al., 2015; Kelly et al., 2016; Lee et al., 2016; Hota et al., 2017; Jiang et al., 2017; Kao et al., 2017; Ianiro et al., 2018; Hvas et al., 2019; Moutinho et al., 2019).

## Effects of FMT on IBD

There are few studies investigating the effectiveness and safety of FMT in IBD in pediatric patients (Nusbaum et al., 2018; Olesen et al., 2020). The medical results are difficult to compare, due to the large discrepancies in the procedure, the small number of respondents, their different ages and the lack of control and placebo groups. FMT was undertaken in patients with Crohn’s disease or ulcerative colitis (UC) (Nusbaum et al., 2018), especially in those who did not respond to standard treatment (Vandenplas et al., 2015; Shimizu et al., 2016; Goyal et al., 2018; Karolewska-Bochenek et al., 2018; Moutinho et al., 2019), and those with concurrent CDI (Hourigan et al., 2015; Cho et al., 2019). The analysis of the available literature shows that the effectiveness of the method, measured in symptom remission, varies. In the case report of 17-year-old male with refractory ulcerative colitis clinical improvement lasted for one month, when symptoms recurred. Second implementation of FMT led to no improvement (Moutinho et al., 2019). In the another case of 11-year-old girl first FMT led to exacerbation of UC symptoms, while repeated procedure allowed her to stay in remission with minimal dose of steroids (Shimizu et al., 2016). An early-onset colitis in an infant girl led to lasting a month improvement and normal defecation (Vandenplas et al., 2015). Good outcomes were observed after two-week FMT course (Karolewska-Bochenek et al., 2018). Clinical remission reached 57% at 1 month and 28% clinical response at 1 and 6 months after FMT (Goyal et al., 2018). A 75% rCDIs cure rate was obtained in patients with IBD three months after FMT (Cho et al., 2019).

### Risk of Adverse Effects

This method appears to be rather well tolerated as the patients had none (Shimizu et al., 2016) or mild to moderate self-limited adverse effects like fever, vomiting, nausea, abdominal pain, bloody stools or diarrhea and flatulence (Goyal et al., 2018; Karolewska-Bochenek et al., 2018; Cho et al., 2019). Yet more severe adverse reactions like tachycardia occurred (Vandenplas et al., 2015). Two patients with recurrence of *C. difficile* underwent colectomy after FMT administration (Cho et al., 2019), however IBD patients are at risk of this procedure due to their condition (Chen et al., 2017). These individuals showed no significant improvement or deterioration in their health from alternative therapy, and the authors believe that the indication for surgery was patients’ condition rather than FMT (Cho et al., 2019).

### The Engraftment of the Donor Microbiota

The engraftment appears to persist for up to 6 months after the procedure, then shifts back to baseline (Hourigan et al., 2015; Goyal et al., 2018). The bacterial diversity restored by the FMT is sustained in children without IBD cured from CDI after single administration (Hourigan et al., 2015). An 8-month maintenance period of the donor GM has even been reported (Shimizu et al., 2016). Repeated FMT appears to be more effective (Shimizu et al., 2016; Karolewska-Bochenek et al., 2018). It is also more effective in patients who have a higher diversity of GM on the baseline, and in those who have lower severity of disease symptoms (Nusbaum et al., 2018).

## Effects of Engraftment of Donor Microbiota FMT on ASD

The growing field of microbial replacement therapies opens up a new pathway to relieve some symptoms previously uncured conditions such as ASDs. In the light of the premise that intestinal and behavioral problems may, at least in part, result from dysbiotic GM and the restoration of eubiosis, may cure some of the GI ailments, and as a secondary outcome, improve some ASD behavioral symptoms, a modified FMT protocol was studied in 18 participants with ASD, aged 7–16 years (Kang et al., 2017). A comprehensive evidence for the effectiveness of this protocol, called microbiota transfer therapy (MTT) in ASDs, has been provided.

The subjects of assessment were dsDNA profile of gut bacteria and bacteriophage and clinical responses. A 2-week vancomycin antibiotic therapy, which has transient therapeutic effects in treating GI symptoms in ASDs (Sandler et al., 2000), followed by bowel cleansing and a high-dose oral or rectal administration of standardized human gut microbiota (SHGM), maintained by lower oral doses for 7–8 weeks, brought about 80% reduction in GI symptoms. An improvement was observed in 89% of participants in diarrhea and constipation, indigestion, and abdominal pain, along with a decrease in the number of days with abnormal stools.

This improvement was retained 8 weeks after therapy (Kang et al., 2017), and the results were evident even in the two years of follow-up (Kang et al., 2019). Another proof for the success of this therapy was the persistence of GM change and the increase in microbial diversity during the second year compared to the end of the original trial, week 18. This confirmed the partial engraftment of the donor microbiota, as well as the preservation of the characteristics and adaptation of the microbiome to the new host, the recipient (Kang et al., 2019). In conclusion, both GI (reduction by 58% after two years versus initial 80%) and ASD behavioral symptoms (47% lower than the baseline versus primary 23%) improved significantly after the therapy, which is encouraging and promising for the treatment of children with ASDs and concurrent GI problems (Kang et al., 2019). The shift of microbiological balance toward the microbiota composition of neurotypical children endorses the hypothesis about the partial causality of abnormal intestinal microbiota in GI symptoms of children with ASDs and the correlation of GI discomfort and behavioral characteristics (Kang et al., 2017).

### Risk of Adverse Effects

MTT seems to be a helpful approach for the alleviation of GI discomfort in children with ASDs, without any adverse negative side effects, but associated with a secondary outcome—improvement of behavioral symptoms (Kang et al., 2019).

## Discussion

An approach that facilitates the complete restoration of gut microbiota in individuals with diseases involving GI symptoms is fecal microbiota transplantation. An advantage of this method over antibiotic or probiotic therapy is that it allows a total exchange of microorganisms in the intestines (Bakken et al., 2011; Gupta et al., 2020), which apart from bacteria, consists of archaea, fungi, and viruses (Cani, 2018), including bacteriophages, which not only regulate the bacterial community (Rascovan et al., 2016), but also impact directly host immune system (Van Belleghem et al., 2018). The viral components of the microbiota is another important but less studied element in the complex network of biological interactions (Handley, 2016). Mycobiome and virome are awaiting in-depth exploration, which will help to achieve a more comprehensive understanding of the role of the microbiome in human health. On the other hand, the disadvantages of this approach are the lack of data on long-term health effects as well as the possibility of disease transmission with the transplanted material.

The mechanism by which the fecal transplant works on treating *C. difficile* (Gupta et al., 2016) infections and alleviating GI disorders (Fang et al., 2018) has not been clearly elucidated. It seems that this approach restores not only the eubiosis, but also possibly the microbial metabolites and components or bacteriophages, as some evidence indicates that when transferred to the recipients, sterile stool filtrates are able to induce a therapeutic effect on CDI symptoms without the potential to generate donor-like microbiota (Ott et al., 2017).The use of SHGM, the main component of which is bacteria (more than 99%) (Kang et al., 2017) is supporting the postulate of eubiosis and perhaps bacterial metabolites in the healing process, at least in the case of ASDs.

However, most of the fecal microbiota transplantation research studies are in adults, not in pediatric patients which account only for 6% (children aged from 0 to 5 years old) and 11% (6 to 17 years old) of all the analysed studies (Olesen et al., 2020). Research on fecal microbiota transplantation, involving children, targets three areas: *C. difficile* infection, IBD and the gut-brain axis (Olesen et al., 2020).

FMT therapy has proven efficacy in the treatment of CDIs; however, it is considered risky for other health reasons. This approach was tested in children with IBD. Evidence suggests that FMT may be useful in this group of disorders but results remain inconclusive. As the findings were obtained on non-heterogeneous groups of patients or in the case-reports, and the procedure of administering the preparation differs practically in each of the cited studies, as well as outcomes measurements.

Due to the similarity of GI symptoms, an attempt was made to test a similar approach for the treatment of ASDs. MTT procedure primarily improves the GI symptoms among ASD patients, and also behavioral symptoms as a secondary outcome (Kang et al., 2017). The authors of the study point to the limitations of the obtained results. The study was conducted with no placebo or blind control, nor randomization. The nature of the control group was based on the observations of the 20 age-matched neurotypical children without the procedure administration. Undoubtedly, individual consideration should be given to determine whether the benefits of this type of therapy outweigh the risks. All these facts show that obtained results should be approached with caution.

The effectiveness and overall safety of the FMT therapy have been proven for rCDIs and shown to be promising for refractory CDIs, while the assessment of its long-term safety is pending (Gupta et al., 2020). The success of FMT can be defined even at the level of 90%, based on the percentage of cases cured of diarrhea associated with rCDIs in the case series (Kassam et al., 2013; van Nood et al., 2013b; Cammarota et al., 2014; Rossen et al., 2015; Gupta et al., 2016). The effectiveness of this therapeutic approach compared to the standard oral vancomycin antibiotic therapy is 81% versus 31% (van Nood et al., 2013b). However, when considering the effectiveness of repeated drug administration and repeated FMT, both the approaches are comparable (Tan and Johnson, 2019).

Among the risk aspects of FMT procedure, its advantage over antibiotic and probiotic is a more comprehensive approach. Intestinal microbiota also consists of other components, which may be as important as bacteria. The effectiveness of probiotic therapy is limited due to the use of bacterial strains known for their medicinal properties and omission of unknown and probably significant factors. However, probiotic supplementation is well tolerated and the serious adverse effects are infrequent, yet rarely evaluated (Liu et al., 2019b). After knowing the microbiota composition of an individual, it may be easier to choose pro- or prebiotic preparations or modify their composition in relation to patient’s needs. NGS technology may be the future standard for this type of diagnostics.

Due to the possibility of transmission of infectious diseases with transplanted material, screening for known pathogens should be performed prior to the FMT procedure, but there are also other risks involved, including the implantation of cancer cells of colon carcinoma (Mullish et al., 2018) or other cancerogenic factors, e.g. HPV virus (Cheng et al., 2019). This event although poorly documented should be taken into account. Besides oral administration, the other routes of administration can cause inconvenience and carry additional risks, ranging from discomfort and adverse reactions to serious medical problems which are rare though possible (e.g. colon perforation in the case of colonoscopy) (Hassan et al., 2016). The FDA’s 2019 statement about FMT warns against the use of colon microbiota transplant procedure due to the confirmed risk of transmission of multidrug-resistant organisms (U.S. Food and Drug Administration, [Accessed 18th June 2020]). The procedure is complicated and heterogeneous at many stages and besides carrying the risk of other diseases, in pediatric patients may cause stress by itself. Limited availability resulting from high costs and complicated logistics underlies the need for a standardized product to effectively treat the patients (Gupta et al., 2020).

### Limitations

This review does not follow a PRISMA methodology. We are aware that we report only two articles describing the modified FMT protocol in ASD, but we are convinced that this may be a promising future direction in treatment of GI in ASD and other neurological disorders.

## Conclusions

Bacteriotherapy based on changing the composition of the intestinal microbiota by transplant of fecal microbiota seem to be a promising tool for treating both GI disturbances and behavioral characteristics associated with ASDs. However, as there are still limited data available on the long-term effects of this therapy, great caution is advised in interpreting the results.

Further studies are necessary, but it can be expected that based on contemporary scientific evidence and experimental research, these kind of therapies will be developed in the future to treat GI disorders in ASDs, which may also have a secondary beneficial effect on behavioral symptoms. Observing the directions of research in neurological disorders, it seems that the FMT approach will become an important research area in the coming years (Vendrik et al., 2020).

## Author Contributions

PŻ conducted the literature search, compiled the information and wrote the manuscript. IŁ contributed academic assistance and edited the manuscript. ŁŁ conceived the article and contributed academic assistance. All authors revised and approved the final version of the manuscript. All authors contributed to the article and approved the submitted version.

## Conflict of Interest

The authors declare that the research was conducted in the absence of any commercial or financial relationships that could be construed as a potential conflict of interest.

## References

[B1] Adams J. B. Johansen L. J. Powell L. D. Quig D. Rubin R. A. (2011). Gastrointestinal flora and gastrointestinal status in children with autism–comparisons to typical children and correlation with autism severity. BMC Gastroenterol. 11, 22. 10.1186/1471-230X-11-22 21410934 PMC3072352

[B2] Agans R. Rigsbee L. Kenche H. Michail S. Khamis H. J. Paliy O. (2011). Distal gut microbiota of adolescent children is different from that of adults. FEMS Microbiol. Ecol. 77, 404–412. 10.1111/j.1574-6941.2011.01120.x 21539582 PMC4502954

[B3] Andreo-Martínez P. García-Martínez N. Sánchez-Samper E. P. Martínez-González A. E. (2020). An approach to gut microbiota profile in children with autism spectrum disorder. Environ. Microbiol. Rep. 12, 115–135. 10.1111/1758-2229.12810 31713352

[B4] Baio J. Wiggins L. Christensen D. L. Maenner M. J. Daniels J. Warren Z.. (2018). Prevalence of Autism Spectrum Disorder Among Children Aged 8 Years - Autism and Developmental Disabilities Monitoring Network, 11 Sites, United State. MMWR Surveill. Summ. 67, 1–23. 10.15585/mmwr.ss6706a1 PMC5919599 29701730

[B5] Bakken J. S. Borody T. Brandt L. J. Brill J. V. Demarco D. C. Franzos M. A.. (2011). Treating Clostridium difficile infection with fecal microbiota transplantation. Clin. Gastroenterol. Hepatol. 9, 1044–1049. 10.1016/j.cgh.2011.08.014 21871249 PMC3223289

[B6] Boem F. Amedei A. (2019). Healthy axis: Towards an integrated view of the gut-brain health. World J. Gastroenterol. 25, 3838–3841. 10.3748/wjg.v25.i29.3838 31413521 PMC6689813

[B7] Borre Y. E. O’Keeffe G. W. Clarke G. Stanton C. Dinan T. G. Cryan J. F. (2014). Microbiota and neurodevelopmental windows: implications for brain disorders. Trends Mol. Med. 20, 509–518. 10.1016/j.molmed.2014.05.002 24956966

[B8] Buie T. Campbell D. B. Fuchs G. J. Furuta G. T. Levy J. Vandewater J.. (2010). Evaluation, diagnosis, and treatment of gastrointestinal disorders in individuals with ASDs: a consensus report. Pediatrics 125 Suppl 1, 1–18. 10.1542/peds.2009-1878C 20048083

[B9] Cammarota G. Ianiro G. Gasbarrini A. (2014). Fecal microbiota transplantation for the treatment of Clostridium difficile infection: a systematic review. J. Clin. Gastroenterol. 48, 693–702. 10.1097/MCG.0000000000000046 24440934

[B10] Cammarota G. Masucci L. Ianiro G. Bibb? S. Dinoi G. Costamagna G.. (2015). Randomised clinical trial: faecal microbiota transplantation by colonoscopy vs. vancomycin for the treatment of recurrent Clostridium difficile infection. Aliment. Pharmacol. Ther. 41, 835–843. 10.1111/apt.13144 25728808

[B11] Cani P. D. (2018). Human gut microbiome: hopes, threats and promises. Gut 67, 1716–1725. 10.1136/gutjnl-2018-316723 29934437 PMC6109275

[B12] Carding S. Verbeke K. Vipond D. T. Corfe B. M. Owen L. J. (2015). Dysbiosis of the gut microbiota in disease. Microb. Ecol. Health Dis. 26, 26191. 10.3402/mehd.v26.26191 25651997 PMC4315779

[B13] Chaidez V. Hansen R. L. Hertz-Picciotto I. (2014). Gastrointestinal problems in children with autism, developmental delays or typical development. J. Autism Dev. Disord. 44, 1117–1127. 10.1007/s10803-013-1973-x 24193577 PMC3981895

[B14] Chen Y. Furuya-Kanamori L. Doi S. A. Ananthakrishnan A. N. Kirk M. (2017). Clostridium difficile Infection and Risk of Colectomy in Patients with Inflammatory Bowel Disease: A Bias-adjusted Meta-analysis. Inflammation Bowel Dis. 23, 200–207. 10.1097/MIB.0000000000000998 28079620

[B15] Cheng Y. W. Phelps E. Ganapini V. Khan N. Ouyang F. Xu H.. (2019). Fecal microbiota transplantation for the treatment of recurrent and severe Clostridium difficile infection in solid organ transplant recipients: A multicenter experience. Am. J. Transplant. 19, 501–511. 10.1111/ajt.15058 30085388 PMC6349556

[B16] Cho I. Blaser M. J. (2012). The human microbiome: at the interface of health and disease. Nat. Rev. Genet. 13, 260–270. 10.1038/nrg3182 22411464 PMC3418802

[B17] Cho S. Spencer E. Hirten R. Grinspan A. Dubinsky M. C. (2019). Fecal Microbiota Transplant for Recurrent Clostridium difficile Infection in Pediatric Inflammatory Bowel Disease. J. Pediatr. Gastroenterol. Nutr. 68, 343–347. 10.1097/MPG.0000000000002172 30320666

[B18] Cryan J. F. O’Riordan K. J. Cowan C. S. M. Sandhu K. V. Bastiaanssen T. F. S. Boehme M.. (2019). The Microbiota-Gut-Brain Axis. Physiol. Rev. 99, 1877–2013. 10.1152/physrev.00018.2018 31460832

[B19] de la Torre-Ubieta L. Won H. Stein J. L. Geschwind D. H. (2016). Advancing the understanding of autism disease mechanisms through genetics. Nat. Med. 22, 345–361. 10.1038/nm.4071 27050589 PMC5072455

[B20] de Magistris L. Familiari V. Pascotto A. Sapone A. Frolli A. Iardino P.. (2010). Alterations of the intestinal barrier in patients with autism spectrum disorders and in their first-degree relatives. J. Pediatr. Gastroenterol. Nutr. 51, 418–424. 10.1097/MPG.0b013e3181dcc4a5 20683204

[B21] Dinan T. G. Cryan J. F. (2017). The Microbiome-Gut-Brain Axis in Health and Disease. Gastroenterol. Clin. North Am. 46, 77–89. 10.1016/j.gtc.2016.09.007 28164854

[B22] D’Cruz A. M. Ragozzino M. E. Mosconi M. W. Shrestha S. Cook E. H. Sweeney J. A. (2013). Reduced behavioral flexibility in autism spectrum disorders. Neuropsychology 27, 152–160. 10.1037/a0031721 23527643 PMC3740947

[B23] Fang H. Fu L. Wang J. (2018). Protocol for Fecal Microbiota Transplantation in Inflammatory Bowel Disease: A Systematic Review and Meta-Analysis. BioMed. Res. Int. 2018, 8941340. 10.1155/2018/8941340 30302341 PMC6158944

[B24] Fattorusso A. Di Genova L. Dell’Isola G. B. Mencaroni E. Esposito S. (2019). Autism Spectrum Disorders and the Gut Microbiota. Nutrients 11(3):521. 10.3390/nu11030521 PMC6471505 30823414

[B25] Feliciano P. Zhou X. Astrovskaya I. Turner T. N. Wang T. Brueggeman L.. (2019). Exome sequencing of 457 autism families recruited online provides evidence for autism risk genes. NPJ Genom. Med. 4, 19. 10.1038/s41525-019-0093-8 31452935 PMC6707204

[B26] Finegold S. M. Downes J. Summanen P. H. (2012). Microbiology of regressive autism. Anaerobe 18, 260–262. 10.1016/j.anaerobe.2011.12.018 22202440

[B27] Foss-Feig J. H. Adkinson B. D. Ji J. L. Yang G. Srihari V. H. McPartland J. C.. (2017). Searching for Cross-Diagnostic Convergence: Neural Mechanisms Governing Excitation and Inhibition Balance in Schizophrenia and Autism Spectrum Disorders. Biol. Psychiatry 81, 848–861. 10.1016/j.biopsych.2017.03.005 28434615 PMC5436134

[B28] Fülling C. Dinan T. G. Cryan J. F. (2019). Gut Microbe to Brain Signaling: What Happens in Vagus. Neuron 101, 998–1002. 10.1016/j.neuron.2019.02.008 30897366

[B29] Garcia-Gutierrez E. Narbad A. Rodr?guez J. M. (2020). Autism Spectrum Disorder Associated With Gut Microbiota at Immune, Metabolomic, and Neuroactive Level. Front. Neurosci. 14, 578666. 10.3389/fnins.2020.578666 33117122 PMC7578228

[B30] Garrett W. S. (2019). The gut microbiota and colon cancer. Science 364, 1133–1135. 10.1126/science.aaw2367 31221845

[B31] Gonzalez A. Stombaugh J. Lozupone C. Turnbaugh P. J. Gordon J. I. Knight R. (2011). The mind-body-microbial continuum. Dialogues Clin. Neurosci. 13, 55–62. 10.31887/DCNS.2011.13.1/agonzalez 21485746 PMC3139398

[B32] Gorrindo P. Williams K. C. Lee E. B. Walker L. S. McGrew S. G. Levitt P. (2012). Gastrointestinal dysfunction in autism: parental report, clinical evaluation, and associated factors. Autism Res. 5, 101–108. 10.1002/aur.237 22511450 PMC3335766

[B33] Goyal A. Yeh A. Bush B. R. Firek B. A. Siebold L. M. Rogers M. B.. (2018). Safety, Clinical Response, and Microbiome Findings Following Fecal Microbiota Transplant in Children With Inflammatory Bowel Disease. Inflamm. Bowel Dis. 24, 410–421. 10.1093/ibd/izx035 29361092

[B34] Gupta A. Khanna S. (2017). Fecal Microbiota Transplantation. JAMA 318, 102. 10.1001/jama.2017.6466 28672320

[B35] Gupta S. Allen-Vercoe E. Petrof E. O. (2016). Fecal microbiota transplantation: in perspective. Therap. Adv. Gastroenterol. 9, 229–239. 10.1177/1756283X15607414 PMC4749851 26929784

[B36] Gupta A. Saha S. Khanna S. (2020). Therapies to modulate gut microbiota: Past, present and future. World J. Gastroenterol. 26, 777–788. 10.3748/wjg.v26.i8.777 32148376 PMC7052537

[B37] Handley S. A. (2016). The virome: a missing component of biological interaction networks in health and disease. Genome Med. 8, 32. 10.1186/s13073-016-0287-y 27037032 PMC4818473

[B38] Hassan M. A. Thomsen C. Vilmann P. (2016). Endoscopic treatment of colorectal perforations–a systematic review. Dan. Med. J. 63(4):A5220. 27034185

[B39] Holingue C. Newill C. Lee L. C. Pasricha P. J. Daniele Fallin M. (2018). Gastrointestinal symptoms in autism spectrum disorder: A review of the literature on ascertainment and prevalence. Autism Res. 11, 24–36. 10.1002/aur.1854 28856868 PMC5773354

[B40] Hota S. S. Sales V. Tomlinson G. Salpeter M. J. McGeer A. Coburn B.. (2017). Oral Vancomycin Followed by Fecal Transplantation Versus Tapering Oral Vancomycin Treatment for Recurrent Clostridium difficile Infection: An Open-Label, Randomized Controlled Trial. Clin. Infect. Dis. 64, 265–271. 10.1093/cid/ciw731 28011612

[B41] Hourigan S. K. Chen L. A. Grigoryan Z. Laroche G. Weidner M. Sears C. L.. (2015). Microbiome changes associated with sustained eradication of Clostridium difficile after single faecal microbiota transplantation in children with and without inflammatory bowel disease. Aliment. Pharmacol. Ther. 42, 741–752. 10.1111/apt.13326 26198180

[B42] Hvas C. L. Dahl J?rgensen S. M. J?rgensen S. P. Storgaard M. Lemming L. Hansen M. M.. (2019). Fecal Microbiota Transplantation Is Superior to Fidaxomicin for Treatment of Recurrent Clostridium difficile Infection. Gastroenterology 156, 1324–1332. 10.1053/j.gastro.2018.12.019 30610862

[B43] Ianiro G. Masucci L. Quaranta G. Simonelli C. Lopetuso L. R. Sanguinetti M.. (2018). Randomised clinical trial: faecal microbiota transplantation by colonoscopy plus vancomycin for the treatment of severe refractory Clostridium difficile infection-single versus multiple infusions. Aliment. Pharmacol. Ther. 48, 152–159. 10.1111/apt.14816 29851107

[B44] Ibrahim S. H. Voigt R. G. Katusic S. K. Weaver A. L. Barbaresi W. J. (2009). Incidence of gastrointestinal symptoms in children with autism: a population-based study. Pediatrics 124, 680–686. 10.1542/peds.2008-2933 19651585 PMC2747040

[B45] Jandhyala S. M. Talukdar R. Subramanyam C. Vuyyuru H. Sasikala M. Nageshwar Reddy D. (2015). Role of the normal gut microbiota. World J. Gastroenterol. 21, 8787–8803. 10.3748/wjg.v21.i29.8787 26269668 PMC4528021

[B46] Jiang Z. D. Ajami N. J. Petrosino J. F. Jun G. Hanis C. L. Shah M.. (2017). Randomised clinical trial: faecal microbiota transplantation for recurrent Clostridum difficile infection - fresh, or frozen, or lyophilised microbiota from a small pool of healthy donors delivered by colonoscopy. Aliment. Pharmacol. Ther. 45, 899–908. 10.1111/apt.13969 28220514

[B47] Jiménez E. Marín M. L. Martín R. Odriozola J. M. Olivares M. Xaus J.. (2008). Is meconium from healthy newborns actually sterile? Res. Microbiol. 159, 187–193. 10.1016/j.resmic.2007.12.007 18281199

[B48] Jyonouchi H. Sun S. Itokazu N. (2002). Innate immunity associated with inflammatory responses and cytokine production against common dietary proteins in patients with autism spectrum disorder. Neuropsychobiology 46, 76–84. 10.1159/000065416 12378124

[B49] Kang D. W. Park J. G. Ilhan Z. E. Wallstrom G. Labaer J. Adams J. B.. (2013). Reduced incidence of Prevotella and other fermenters in intestinal microflora of autistic children. PloS One 8, e68322. 10.1371/journal.pone.0068322 23844187 PMC3700858

[B50] Kang V. Wagner G. C. Ming X. (2014). Gastrointestinal dysfunction in children with autism spectrum disorders. Autism Res. 7, 501–506. 10.1002/aur.1386 24753336

[B51] Kang D. W. Adams J. B. Gregory A. C. Borody T. Chittick L. Fasano A.. (2017). Microbiota Transfer Therapy alters gut ecosystem and improves gastrointestinal and autism symptoms: an open-label study. Microbiome 5, 10. 10.1186/s40168-016-0225-7 28122648 PMC5264285

[B52] Kang D. W. Adams J. B. Coleman D. M. Pollard E. L. Maldonado J. McDonough-Means S.. (2019). Long-term benefit of Microbiota Transfer Therapy on autism symptoms and gut microbiota. Sci. Rep. 9, 5821. 10.1038/s41598-019-42183-0 30967657 PMC6456593

[B53] Kao D. Roach B. Silva M. Beck P. Rioux K. Kaplan G. G.. (2017). Effect of Oral Capsule- vs Colonoscopy-Delivered Fecal Microbiota Transplantation on Recurrent Clostridium difficile Infection: A Randomized Clinical Trial. JAMA 318, 1985–1993. 10.1001/jama.2017.17077 29183074 PMC5820695

[B54] Karolewska-Bochenek K. Grzesiowski P. Banaszkiewicz A. Gawronska A. Kotowska M. Dziekiewicz M.. (2018). A Two-Week Fecal Microbiota Transplantation Course in Pediatric Patients with Inflammatory Bowel Disease. Adv. Exp. Med. Biol. 1047, 81–87. 10.1007/5584_2017_123 29151253

[B55] Kassam Z. Lee C. H. Yuan Y. Hunt R. H. (2013). Fecal microbiota transplantation for Clostridium difficile infection: systematic review and meta-analysis. Am. J. Gastroenterol. 108, 500–508. 10.1038/ajg.2013.59 23511459

[B56] Kelly C. R. Khoruts A. Staley C. Sadowsky M. J. Abd M. Alani M.. (2016). Effect of Fecal Microbiota Transplantation on Recurrence in Multiply Recurrent Clostridium difficile Infection: A Randomized Trial. Ann. Intern. Med. 165, 609–616. 10.7326/M16-0271 27547925 PMC5909820

[B57] Kostic A. D. Xavier R. J. Gevers D. (2014). The microbiome in inflammatory bowel disease: current status and the future ahead. Gastroenterology 146, 1489–1499. 10.1053/j.gastro.2014.02.009 24560869 PMC4034132

[B58] Kuijper E. J. Coignard B. T?ll P. (2006). Emergence of Clostridium difficile-associated disease in North America and Europe. Clin. Microbiol. Infect. 12 Suppl 6, 2–18. 10.1111/j.1469-0691.2006.01580.x 16965399

[B59] Łaczmańska I. Złocińska M. Kozłowska J. Skiba P. Pesz K. Slezak R.. (2020). Multiplex ligation-dependent probe amplification as a screening test in children with autism spectrum disorders. Adv. Clin. Exp. Med. 29 (1), 101–106. 10.17219/acem/112609 31990460

[B60] Lederberg J. McCray A. T. (2001). ‘O’Ome Sweet ‘Omics–A Genealogical Treasury of Words. Sci., 8.

[B61] Lee C. H. Steiner T. Petrof E. O. Smieja M. Roscoe D. Nematallah A.. (2016). Frozen vs Fresh Fecal Microbiota Transplantation and Clinical Resolution of Diarrhea in Patients With Recurrent Clostridium difficile Infection: A Randomized Clinical Trial. JAMA 315, 142–149. 10.1001/jama.2015.18098 26757463

[B62] Liu F. Li J. Wu F. Zheng H. Peng Q. Zhou H. (2019a). Altered composition and function of intestinal microbiota in autism spectrum disorders: a systematic review. Transl. Psychiatry 9, 43. 10.1038/s41398-019-0389-6 30696816 PMC6351640

[B63] Liu J. Wan G. B. Huang M. S. Agyapong G. Zou T. L. Zhang X. Y.. (2019b). Probiotic Therapy for Treating Behavioral and Gastrointestinal Symptoms in Autism Spectrum Disorder: A Systematic Review of Clinical Trials. Curr. Med. Sci. 39, 173–184. 10.1007/s11596-019-2016-4 31016507

[B64] Lozupone C. A. Stombaugh J. I. Gordon J. I. Jansson J. K. Knight R. (2012). Diversity, stability and resilience of the human gut microbiota. Nature 489, 220–230. 10.1038/nature11550 22972295 PMC3577372

[B65] Macfabe D. F. (2012). Short-chain fatty acid fermentation products of the gut microbiome: implications in autism spectrum disorders. Microb. Ecol. Health Dis. 23:19260. 10.3402/mehd.v23i0.19260 PMC3747729 23990817

[B66] MacFabe D. F. (2015). Enteric short-chain fatty acids: microbial messengers of metabolism, mitochondria, and mind: implications in autism spectrum disorders. Microb. Ecol. Health Dis. 26, 28177. 10.3402/mehd.v26.28177 26031685 PMC4451098

[B67] Maenner M. J. Shaw K. A. Baio J. Washington A. Patrick M. DiRienzo M.. (2020). Prevalence of Autism Spectrum Disorder Among Children Aged 8 Years - Autism and Developmental Disabilities Monitoring Network, 11 Sites, United States 2016. MMWR Surveill. Summ. 69, 1–12. 10.15585/mmwr.ss6904a1 PMC7119644 32214087

[B68] Makino H. Kushiro A. Ishikawa E. Muylaert D. Kubota H. Sakai T.. (2011). Transmission of intestinal Bifidobacterium longum subsp. longum strains from mother to infant, determined by multilocus sequencing typing and amplified fragment length polymorphism. *Appl. Environ*. Microbiol 77, 6788–6793. 10.1128/AEM.05346-11 PMC3187114 21821739

[B69] Malik T. A. (2015). Inflammatory Bowel Disease: Historical Perspective, Epidemiology, and Risk Factors. Surg. Clin. North Am. 95, 1105–1122. 10.1016/j.suc.2015.07.006 26596917

[B70] Martín R. Langa S. Reviriego C. Jiménez E. Marín M. L. Xaus J.. (2003). Human milk is a source of lactic acid bacteria for the infant gut. J. Pediatr. 143, 754–758. 10.1016/j.jpeds.2003.09.028 14657823

[B71] Martínez-González A. E. Andreo-Martínez P. (2019). The Role of Gut Microbiota in Gastrointestinal Symptoms of Children with ASD. Med. (Kaunas) 55 (8), 408. 10.3390/medicina55080408 PMC6722942 31357482

[B72] Martínez-González A. E. Andreo-Martínez P. (2020). Prebiotics, probiotics and fecal microbiota transplantation in autism: A systematic review. Rev. Psiquiatr. Salud. Ment. 13, 150–164. 10.1016/j.rpsm.2020.06.002 32684346

[B73] McDonald L. C. Gerding D. N. Johnson S. Bakken J. S. Carroll K. C. Coffin S. E.. (2018). Clinical Practice Guidelines for Clostridium difficile Infection in Adults and Children: 2017 Update by the Infectious Diseases Society of America (IDSA) and Society for Healthcare Epidemiology of America (SHEA). Clin. Infect. Dis. 66, e1–e48. 10.1093/cid/cix1085 29462280 PMC6018983

[B74] McElhanon B. O. McCracken C. Karpen S. Sharp W. G. (2014). Gastrointestinal symptoms in autism spectrum disorder: a meta-analysis. Pediatrics 133, 872–883. 10.1542/peds.2013-3995 24777214

[B75] Moutinho B. D. Baima J. P. Rigo F. F. Saad-Hossne R. Rodrigues J. Romeiro F. G.. (2019). Fecal microbiota transplantation in refractory ulcerative colitis - a case report. J. Int. Med. Res. 47, 1072–1079. 10.1177/0300060518821790 30632438 PMC6381481

[B76] Mullish B. H. Quraishi M. N. Segal J. P. McCune V. L. Baxter M. Marsden G. L.. (2018). The use of faecal microbiota transplant as treatment for recurrent or refractory Clostridium difficile infection and other potential indications: joint British Society of Gastroenterology (BSG) and Healthcare Infection Society (HIS) guidelines. Gut 67, 1920–1941. 10.1136/gutjnl-2018-316818 30154172

[B77] Navarro F. Liu Y. Rhoads J. M. (2016). Can probiotics benefit children with autism spectrum disorders? World J. Gastroenterol. 22, 10093–10102. 10.3748/wjg.v22.i46.10093 28028357 PMC5155168

[B78] Nusbaum D. J. Sun F. Ren J. Zhu Z. Ramsy N. Pervolarakis N.. (2018). Gut microbial and metabolomic profiles after fecal microbiota transplantation in pediatric ulcerative colitis patients. FEMS Microbiol. Ecol. 94 (9), fiy133. 10.1093/femsec/fiy133 PMC6454419 30010747

[B79] Olesen S. W. Panchal P. Chen J. Budree S. Osman M. (2020). Global disparities in faecal microbiota transplantation research. Lancet Gastroenterol. Hepatol. 5, 241. 10.1016/S2468-1253(19)30452-2 32061326

[B80] Ott S. J. Waetzig G. H. Rehman A. Moltzau-Anderson J. Bharti R. Grasis J. A.. (2017). Efficacy of Sterile Fecal Filtrate Transfer for Treating Patients With Clostridium difficile Infection. Gastroenterology 152, 799–811. 10.1053/j.gastro.2016.11.010 27866880

[B81] Pashankar D. S. (2005). Childhood constipation: evaluation and management. Clin. Colon. Rectal. Surg. 18, 120–127. 10.1055/s-2005-870894 20011352 PMC2780136

[B82] Peeters B. Noens I. Philips E. M. Kuppens S. Benninga M. A. (2013). Autism spectrum disorders in children with functional defecation disorders. J. Pediatr. 163, 873–878. 10.1016/j.jpeds.2013.02.028 23522863

[B83] Pulikkan J. Mazumder A. Grace T. (2019). Role of the Gut Microbiome in Autism Spectrum Disorders. Adv. Exp. Med. Biol. 1118, 253–269. 10.1007/978-3-030-05542-4_13 30747427

[B84] Pusponegoro H. D. Ismael S. Sastroasmoro S. Firmansyah A. Vandenplas Y. (2015). Maladaptive Behavior and Gastrointestinal Disorders in Children with Autism Spectrum Disorder. Pediatr. Gastroenterol. Hepatol. Nutr. 18, 230–237. 10.5223/pghn.2015.18.4.230 26770897 PMC4712535

[B85] Raffaele A. Vatta F. Votto M. Licari A. Ruffoli M. Brunero M.. (2021). Eosinophilic colitis in children: a new and elusive enemy? Pediatr. Surg. Int. 10.1007/s00383-020-04832-8 33409540

[B86] Rao K. Safdar N. (2016). Fecal microbiota transplantation for the treatment of Clostridium difficile infection. J. Hosp. Med. 11, 56–61. 10.1002/jhm.2449 26344412 PMC4908581

[B87] Rascovan N. Duraisamy R. Desnues C. (2016). Metagenomics and the Human Virome in Asymptomatic Individuals. Annu. Rev. Microbiol. 70, 125–141. 10.1146/annurev-micro-102215-095431 27607550

[B88] Ringel-Kulka T. Cheng J. Ringel Y. Salojärvi J. Carroll I. Palva A.. (2013). Intestinal microbiota in healthy U.S. young children and adults–a high throughput microarray analysis. PloS One 8, e64315. 10.1371/journal.pone.0064315 23717595 PMC3662718

[B89] Rogowska A. (2012). Eozynofilowe choroby przewodu pokarmowego. Gastroenterol. Kliniczna. Postepy i Standardy 4, 105–116.

[B90] Rose D. R. Yang H. Serena G. Sturgeon C. Ma B. Careaga M.. (2018). Differential immune responses and microbiota profiles in children with autism spectrum disorders and co-morbid gastrointestinal symptoms. Brain Behav. Immun. 70, 354–368. 10.1016/j.bbi.2018.03.025 29571898 PMC5953830

[B91] Rossen N. G. MacDonald J. K. de Vries E. M. D’Haens G. R. de Vos W. M. Zoetendal E. G.. (2015). Fecal microbiota transplantation as novel therapy in gastroenterology: A systematic review. World J. Gastroenterol. 21, 5359–5371. 10.3748/wjg.v21.i17.5359 25954111 PMC4419078

[B92] Sandler R. H. Finegold S. M. Bolte E. R. Buchanan C. P. Maxwell A. P. Väisänen M. L.. (2000). Short-term benefit from oral vancomycin treatment of regressive-onset autism. J. Child Neurol. 15, 429–435. 10.1177/088307380001500701 10921511

[B93] Sandoval-Motta S. Aldana M. Martínez-Romero E. Frank A. (2017). The Human Microbiome and the Missing Heritability Problem. Front. Genet. 8, 80. 10.3389/fgene.2017.00080 28659968 PMC5468393

[B94] Santocchi E. Guiducci L. Fulceri F. Billeci L. Buzzigoli E. Apicella F.. (2016). Gut to brain interaction in Autism Spectrum Disorders: a randomized controlled trial on the role of probiotics on clinical, biochemical and neurophysiological parameters. BMC Psychiatry 16, 183. 10.1186/s12888-016-0887-5 27260271 PMC4893248

[B95] Satokari R. Grönroos T. Laitinen K. Salminen S. Isolauri E. (2009). Bifidobacterium and Lactobacillus DNA in the human placenta. Lett. Appl. Microbiol. 48, 8–12. 10.1111/j.1472-765X.2008.02475.x 19018955

[B96] Saurman V. Margolis K. G. Luna R. A. (2020). Autism Spectrum Disorder as a Brain-Gut-Microbiome Axis Disorder. Dig. Dis. Sci. 65, 818–828. 10.1007/s10620-020-06133-5 32056091 PMC7580230

[B97] Scriven M. Dinan T. G. Cryan J. F. Wall M. (2018). Neuropsychiatric Disorders: Influence of Gut Microbe to Brain Signalling. Diseases 6, 78. 10.3390/diseases6030078 PMC6163507 30200574

[B98] Sekirov I. Russell S. L. Antunes L. C. Finlay B. B. (2010). Gut microbiota in health and disease. Physiol. Rev. 90, 859–904. 10.1152/physrev.00045.2009 20664075

[B99] Shafquat A. Joice R. Simmons S. L. Huttenhower C. (2014). Functional and phylogenetic assembly of microbial communities in the human microbiome. Trends Microbiol. 22, 261–266. 10.1016/j.tim.2014.01.011 24618403 PMC4008634

[B100] Shanahan F. (2012). The gut microbiota-a clinical perspective on lessons learned. Nat. Rev. Gastroenterol. Hepatol. 9, 609–614. 10.1038/nrgastro.2012.145 22890109

[B101] Shimizu H. Arai K. Abe J. Nakabayashi K. Yoshioka T. Hosoi K.. (2016). Repeated fecal microbiota transplantation in a child with ulcerative colitis. Pediatr. Int. 58, 781–785. 10.1111/ped.12967 27324973

[B102] Srikantha P. Mohajeri M. H. (2019). The Possible Role of the Microbiota-Gut-Brain-Axis in Autism Spectrum Disorder. Int. J. Mol. Sci. 20(9), 2115. 10.3390/ijms20092115 PMC6539237 31035684

[B103] Tan X. Johnson S. (2019). Fecal microbiota transplantation (FMT) for C. difficile infection, just say ‘No’. Anaerobe 60, 102092. 10.1016/j.anaerobe.2019.102092 31472233

[B104] Turnbaugh P. J. Ley R. E. Hamady M. Fraser-Liggett C. M. Knight R. Gordon J. I. (2007). The human microbiome project. Nature 449, 804–810. 10.1038/nature06244 17943116 PMC3709439

[B105] U. S. Food and Drug Administration. FDA In Brief: FDA warns about potential risk of serious infections caused by multi-drug resistant organisms related to the investigational use of Fecal Microbiota for Transplantation (Accessed 18th June 2020).

[B106] U. S. Food and Drug Administration. U.S. Food and Drug Administration. Enforcement Policy Regarding Investigational New Drug Requirements for Use of Fecal Microbiota for Transplantation to Treat Clostridium difficile Infection Not Responsive to Standard Therapies. Available at: https://www.fda.gov/media/86440/download (Accessed May 22, 2020).

[B107] Van Belleghem J. D. Dabrowska K. Vaneechoutte M. Barr J. J. Bollyky P. L. (2018). Interactions between Bacteriophage, Bacteria, and the Mammalian Immune System. Viruses 11 (1), 10. 10.3390/v11010010 PMC6356784 30585199

[B108] van Nood E. Dijkgraaf M. G. Keller J. J. (2013a). Duodenal infusion of feces for recurrent Clostridium difficile. N. Engl. J. Med. 368, 2145. 10.1056/NEJMc1303919 23718168

[B109] van Nood E. Vrieze A. Nieuwdorp M. Fuentes S. Zoetendal E. G. de Vos W. M.. (2013b). Duodenal infusion of donor feces for recurrent Clostridium difficile. N. Engl. J. Med. 368, 407–415. 10.1056/NEJMoa1205037 23323867

[B110] Vandenplas Y. Veereman G. van der Werff Ten Bosch J. Goossens A. Pierard D. Samsom J. N.. (2015). Fecal Microbial Transplantation in Early-Onset Colitis: Caution Advised. J. Pediatr. Gastroenterol. Nutr. 61, e12–e14. 10.1097/MPG.0000000000000281 24399213

[B111] Vendrik K. E. W. Ooijevaar R. E. de Jong P. R. C. Laman J. D. van Oosten B. W. van Hilten J. J.. (2020). Fecal Microbiota Transplantation in Neurological Disorders. Front. Cell Infect. Microbiol. 10, 98. 10.3389/fcimb.2020.00098 32266160 PMC7105733

[B112] Walker S. J. Fortunato J. Gonzalez L. G. Krigsman A. (2013). Identification of unique gene expression profile in children with regressive autism spectrum disorder (ASD) and ileocolitis. PloS One 8, e58058. 10.1371/journal.pone.0058058 23520485 PMC3592909

[B113] Wang B. Yao M. Lv L. Ling Z. Li L. (2017). The human microbiota in health and disease. Engineering 3, 71 – 82. 10.1016/J.ENG.2017.01.008

[B114] Wang J. W. Kuo C. H. Kuo F. C. Wang Y. K. Hsu W. H. Yu F. J.. (2019a). Fecal microbiota transplantation: Review and update. J. Formos. Med. Assoc. 118 Suppl 1, S23–S31. 10.1016/j.jfma.2018.08.011 30181015

[B115] Wang M. Zhou J. He F. Cai C. Wang H. Wang Y.. (2019b). Alteration of gut microbiota-associated epitopes in children with autism spectrum disorders. Brain Behav. Immun. 75, 192–199. 10.1016/j.bbi.2018.10.006 30394313

[B116] Wopereis H. Oozeer R. Knipping K. Belzer C. Knol J. (2014). The first thousand days - intestinal microbiology of early life: establishing a symbiosis. Pediatr. Allergy Immunol. 25, 428–438. 10.1111/pai.12232 24899389

[B117] World Health Organization. (2019). FDA In Brief: FDA warns about potential risk of serious infections caused by multi-drug resistant organisms related to the investigational use of Fecal Microbiota for TransplantationWorld Health Organisation, Autism spectrum disorders. [Online]. Available at: https://www.who.int/news-room/fact-sheets/detail/autism-spectrum-disorders (Accessed 14th April 2020).

[B118] Xu M. Xu X. Li J. Li F. (2019). Association Between Gut Microbiota and Autism Spectrum Disorder: A Systematic Review and Meta-Analysis. Front. Psychiatry 10, 473. 10.3389/fpsyt.2019.00473 31404299 PMC6673757

[B119] Yatsunenko T. Rey F. E. Manary M. J. Trehan I. Dominguez-Bello M. G. Contreras M.. (2012). Human gut microbiome viewed across age and geography. Nature 486, 222–227. 10.1038/nature11053 22699611 PMC3376388

[B120] Youngster I. Sauk J. Pindar C. Wilson R. G. Kaplan J. L. Smith M. B.. (2014). Fecal microbiota transplant for relapsing Clostridium difficile infection using a frozen inoculum from unrelated donors: a randomized, open-label, controlled pilot study. Clin. Infect. Dis. 58, 1515–1522. 10.1093/cid/ciu135 24762631 PMC4017893

